# Randomized comparison between a forced air system and warm water bath for resuscitation of neonatal hypothermic calves with or without oral administration of caffeine

**DOI:** 10.1111/jvim.17066

**Published:** 2024-04-29

**Authors:** Adam T. Copeland, Amanda J. Kreuder, Grant Dewell, Renee Dewell, Caitlin Wiley, Lingnan Yuan, Jonathan P. Mochel, Joe S. Smith

**Affiliations:** ^1^ Department of Veterinary Diagnostic and Production Animal Medicine, College of Veterinary Medicine Iowa State University Ames Iowa USA; ^2^ Department of Veterinary Microbiology and Preventive Medicine, College of Veterinary Medicine Iowa State University Ames Iowa USA; ^3^ The Center for Food Security and Public Health, College of Veterinary Medicine Iowa State University Ames Iowa USA; ^4^ Department of Biomedical Sciences, College of Veterinary Medicine Iowa State University Ames Iowa USA; ^5^ Large Animal Clinical Sciences, College of Veterinary Medicine University of Tennessee Knoxville Tennessee USA

**Keywords:** 5‐hour energy, cortisol, hypothermia, neonates, rewarming

## Abstract

**Background:**

Hypothermia is a cause of neonatal calf death in cold climates. Practical and effective rewarming methods are important for bovine health within affected regions.

**Hypothesis/Objectives:**

To compare the rewarming rate and blood analytes (glucose, lactate, and cortisol) of calves resuscitated with forced air with warm water bath, with or without oral administration of caffeine.

**Animals:**

Twenty healthy neonatal Holstein bull calves.

**Methods:**

In this randomized, prospective study, calves born healthy and without history of dystocia were cooled to 32°C rectal temperature then thermally resuscitated using either forced air rewarming or warm water bath (40°C) with or without oral administration of caffeine. Rectal temperatures were used to quantify recovery rate. Measurements of glucose, lactate, and cortisol were recorded for every 2°C change in rectal temperature.

**Results:**

Rectal temperature decline (0.03°C per minute) and total cooling time (191.0 ± 33.3 minutes) did not significantly differ among treatment groups. Calves were successfully resuscitated to 38°C by either method. Time required to euthermia using warm water was significantly faster (0.1°C per minute; 64.3 ± 17.8 minute; *P* < .05) than forced air (0.05°C per minute; 123.1 ± 20.0 minutes). Caffeine had no significant effect on resuscitation rate (*P* = .14; 95% CI, −0.002 to 0.024) in either treatment; however, caffeine was associated with reduced time to euthermia by 8.3 and 10.8 minutes, respectively. Changes in metabolic variables (glucose, lactate, and cortisol), were inversely related to rectal temperature with no statistical significance among rewarming methods.

**Conclusions and Clinical Importance:**

Although warm water submersion is faster, forced air rewarming is an effective alternative for restoration of euthermia.

**Abbreviations**
C: celsius
G: gram
L: liter

## 1 INTRODUCTION

Thermoregulation is essential during the perinatal period (birth to 48 hours) when energy demands are high, and neonates must rapidly acclimate to extrauterine temperatures. A large surface area : mass ratio, low percentage of white adipose tissue, and reduced shivering predispose neonates to heat loss.^1^ The rudimentary forestomach of ruminant neonates precludes the utilization of heat derived from microbial fermentation as in mature ruminants.^2^ When ambient temperatures decrease below the lower critical thermal limit, defined as the point at which metabolic heat production increases to maintain core body temperature (approximately 13°C^3^), the neonates must rely on nutrition and stored fat as substrates for maintenance of normal body temperature. The average bovine calf is born with an estimated 18 hours of energy reserves in the form of 380 to 400‐g adipose tissue and 180‐g glycogen,^4^ making them more cold‐tolerant than most other species.^3^ However, any additional thermal stressor increases these metabolic requirements and might lead to complete energy reserve depletion and hypothermia.^1^, ^3^, ^5^ Accidental hypothermia‐induced central nervous system depression, cardiac dysrhythmias, and reduced tissue oxygenation^6^ predispose cold‐stressed ruminant neonates to acute death or delayed mortality from failure of transfer of passive immunity related conditions. These weather‐related losses alone comprise 13.7% of all nonpredator calf deaths in the United States and warrant investigation to mitigate the associated $100 million in lost annual revenue.^7^

Warm water baths have long been considered superior to heating pads, infrared lamps, or blankets for hypothermic resuscitation of calves because of a faster return to euthermia with reduced energy expenditure.^8^, ^9^, ^10^ Unfortunately, the practicality of this process renders it challenging for on‐farm use and has been replaced by commercial forced air delivery systems. Indirect heat modulation has also been attempted with mixed results through independent or adjunct IV administration of glucose, oral administration of colostrum^11^ or ethanol,^8^ and prepartum dam fat supplementation.^12^, ^13^ In addition, oral administration of caffeine supplements, most notably 5‐hour ENERGY (Living Essentials LLC, Farmington Hills, Michigan, USA), has recently gained popularity in the midwestern United States after anecdotal reports advocated such products to hypothermic or otherwise weak bovine calves.^14^ Current literature suggests caffeine can upregulate nonshivering thermogenesis in various animal models^15^, ^16^, ^17^, ^18^, ^19^; however, this has not been documented in calves.

The objectives of this study were to (i) compare the time to euthermia among hypothermic bovine neonatal calves resuscitated in a commercial forced air rewarming unit versus warm water bath of equal temperature, (ii) evaluate the adjunct effect of PO administered caffeine supplement on the time to euthermia, and (iii) characterize changes in glucose, lactate, and cortisol, during neonatal calf hypothermia development as well as any differences induced by the rewarming methods. We hypothesized that the warm water bath would resuscitate the rectal temperature of hypothermic calves faster than the forced air delivery systems, the oral caffeine supplement would have no significant effect on time to euthermia, and blood analytes would increase throughout the cooling process then return to baseline once euthermic.

## 2 MATERIALS AND METHODS

This study was carried out at the Farm Animal and Camelid Hospital, Iowa State University, College of Veterinary Medicine. All methods and materials described were in accordance with institutional guidelines outlined in the Iowa State University Institutional Animal Care and Use Committee (IACUC protocol 19‐252).

### 2.1 Animals

Twenty Holstein bull calves (<24 hours old, body weight 41.6 ± 4.3 kg) born without history of dystocia and deemed healthy upon veterinary inspection were enrolled in this study. All calves were sourced from a private Central Iowa−based owner and identically processed before shipment in accordance with the protocols of the source dairy. Briefly, all calves were individually identified with ear tags, had their navels dipped in povidone iodine, fed 3.8 L of pasteurized colostrum, and received immunization or passive antibody protection against the following infectious agents: bovine rotavirus and coronavirus, *Clostridium perfringens* type C and D, and *C. tetani*. Enrolled animals were transported from the farm in an enclosed trailer to a climate‐controlled (25°C) environment at Iowa State University College of Veterinary Medicine within the first 24 hours of life. Calves were acclimated to their new environment for 12 hours before the study's onset. Calves were fed commercial milk replacer twice daily in accordance with National Research Council–recommended guidelines and provided access to water ad libitum. Calves were housed in groups of 5 in 14‐m^2^ pens with opportunity for calf‐to‐calf contact.

### 2.2 Experimental design

Upon enrollment, animals were randomly allocated to 4 different treatment groups or a negative control group with a commercial randomization program (Excel, Microsoft Corporation, Redmond, Washington, USA): forced air, forced air with oral caffeine supplement, warm water bath, warm water bath with oral caffeine supplement, or a negative control group. A positive control group was not included in the study design because hypothermic calves deprived of thermal support were deemed likely to experience considerable negative outcomes and acquire undesirable comorbidities while minimally contributing to the study objectives.

### 2.3 Cooling procedure

A thermostat‐regulated water immersion system was constructed by a modified version of the protocol described in Robinson and Young, 1988.^8^ Before the cooling procedure, a 14‐gauge 9‐cm polyurethane IV catheter (MILA International, Florence, Kentucky) was aseptically placed in the jugular vein of all calves and sutured to the skin after attachment of a high flow extension set (MILA International, Florence, Kentucky, USA). Calves were then acclimated to the restraint and floating sensation while in a 38°C water bath for 20 minutes. This was accomplished by restraining calves in sternal recumbency while suspended in a 0.6 m × 0.6 m × 1.2 m galvanized water tank with the calf's head and neck unsubmerged. After the 20‐minute acclimation period, the calf and restraint apparatus were simultaneously transferred to a second tank containing 18°C water and equipped with a circulating pump (FREESEA 160‐1100, Freesea Industrial Co, Guangzhou, Guangdong, PRC) until a rectal temperature of 32°C was achieved. If the calf's rectal temperature did not reach 32°C within 2 hours, the water temperature was further reduced to 10°C until the end point rectal temperature was achieved. Control animals were left at room temperature and unexposed to the cooling process.

### 2.4 Rewarming procedure

Animals were removed from the cooling apparatus upon reaching a rectal temperature of 32°C. Calves assigned to forced air rewarming groups were manually removed from the restraint apparatus, towel dried, and placed on a grate in a commercial warming box (PolyDome Calf Warmer, Litchfield, Minnesota, USA) in <1 minute. The air temperature within the warming box was regulated at 40°C (±1°C) with a thermostat switch (ITC308S, Inkbird, Shenzhen, Guangdong, PRC). Calves rewarmed in the warm water bath were unaltered and transferred in the same restraint apparatus used previously into a prewarmed galvanized tank filled with 40°C (±1°C) circulating water regulated by 2 bucket warmers (Marshalltown Bucket Heater 742G, Marshalltown, Iowa, USA) and a thermostat. Calves designated to receive the caffeine supplement were administered the entire contents of a single 57‐mL 5‐hour ENERGY (Living Essentials LLC, Farmington Hills, Michigan, USA) bottle containing 230‐mg caffeine via esophageal intubation before rewarming initiation. The rewarming period was considered complete once the rectal temperature reached 38°C. Upon removal from their respective treatment environments, all calves received manual towel drying and povidone iodine naval dipping before returning to group housing at room temperature for 24 hours of observation thereafter. Control animals were left at room temperature and were unexposed to the rewarming process.

### 2.5 Measurements and sample collection

A calibrated rectal thermometer (GLA‐M500, GLA Agricultural Electronics, San Luis Obispo, California, USA), inserted to a depth of 11.5 cm, recorded rectal temperatures throughout the study to characterize the change rate in response to the treatment. Rectal temperatures were recorded at least once every 20 minutes during the cooling procedure; more frequent temperature monitoring occurred when nearing each desired rectal temperatures (36°C, 34°C, and 32°C) to ensure punctual sample collection. After placement in the respective rewarming environment, rectal temperatures were recorded every 10 minutes until euthermic (38°C). The perineal region was lifted above the water line before thermometer probe insertion, when necessary, to reduce artificial readings associated with incidental water enemas. Because control animals were not subjected to the cooling or rewarming process, blood collection timing coincided with a randomly paired treatment animal. This permitted analyte evaluation over a similar time frame as the rewarmed calves. In short, blood was collected from the paired calves each time the treatment animal reached the desired rectal temperature.

Initially, 8 mL of blood was collected via the jugular catheter before placement within the 18°C cooling bath, followed by every 2°C change in rectal temperature thereafter until conclusion of the study by a modified approach to the push‐pull method.^20^ Briefly, manufacturer‐reported priming volumes were used to estimate a combined dead space of 1.35 mL within the polyurethane catheter and extension set. Then, 6 mL of blood (>300% of dead space calculation) was aspirated and pushed back into the catheter with a sterile 12‐mL syringe directly connected to the high flow extension. This process was repeated 3 times before a final 8‐mL sample was collected. Whole blood glucose (Alpha Trak2, Zoetis, Parsippany, New Jersey, USA) and lactate (Lactate Plus, Nova Biomedical, Waltham, Massachusetts, USA) were analyzed before placing the remaining blood sample in a sodium heparin tube and stored on a 4°C ice bath until spun (1500×*g* for 10 minutes). Plasma was then pipetted into cryovials and stored at −80°C until analyzed for cortisol concentrations via coated tube radioimmunoassay (MP Biologics, Kit # 07‐221105R).

### 2.6 Statistical analysis

Sample size was chosen based on published studies of similar design with 4 animals allocated to treatment groups.^8^, ^9^ Twenty calves, each with respective weights and varied time intervals among 7 blood samples corresponding to blood glucose, lactate, and cortisol concentrations at desired rectal temperatures, were included in the analysis. The multivariate linear regression was conducted by R 3.5.2 to assess the difference in the change rate of rectal temperature, glucose, lactate, and cortisol among forced air, water bath, and caffeine supplement administration. The main effects of time and treatment groups and the interaction between time and groups are included in the linear model. The 95% confidence intervals (CIs) of time effects in different treatment groups were calculated. If the confidence intervals did not overlap with each other, the corresponding treatment groups were considered significantly different. If the confidence interval contained 0, the time effect of corresponding treatment group was considered significant. The effect of weight on the aforementioned variables was also assessed. *P*‐values < .05 and nonoverlap between confidence intervals were considered significant. Descriptive analysis of time and afterdrop, as well as all figures, were made by GraphPad Prism (GraphPad Software, San Diego, California, USA). Shivering and resistance to submersion were evaluated as binary categorical values with options of present or not present.

## 3 RESULTS

All calves tolerated being submerged in a sternal recumbent position within a cloth cradle during the acclimation period. Intermittent episodes (<1 minute) of struggling ensued after submersion in the cooling bath and ceased within approximately 10 minutes into the cooling procedure. Shivering was noted in all calves once rectal temperatures reached 36°C and persisted until euthermic. In addition, all subjects were obtunded at extrication from the cooling process with severe head and neck muscle rigidity, making esophageal tubing difficult. Calves rewarmed in the air chamber were observed having intermittent bursts of erratic and uncoordinated movements especially when stimulated during rectal temperature measurements or manually adjusted in the chamber. Although not quantifiably measured, these episodes seemed to overall decrease in frequency as the subjects warmed.

**FIGURE 1:**
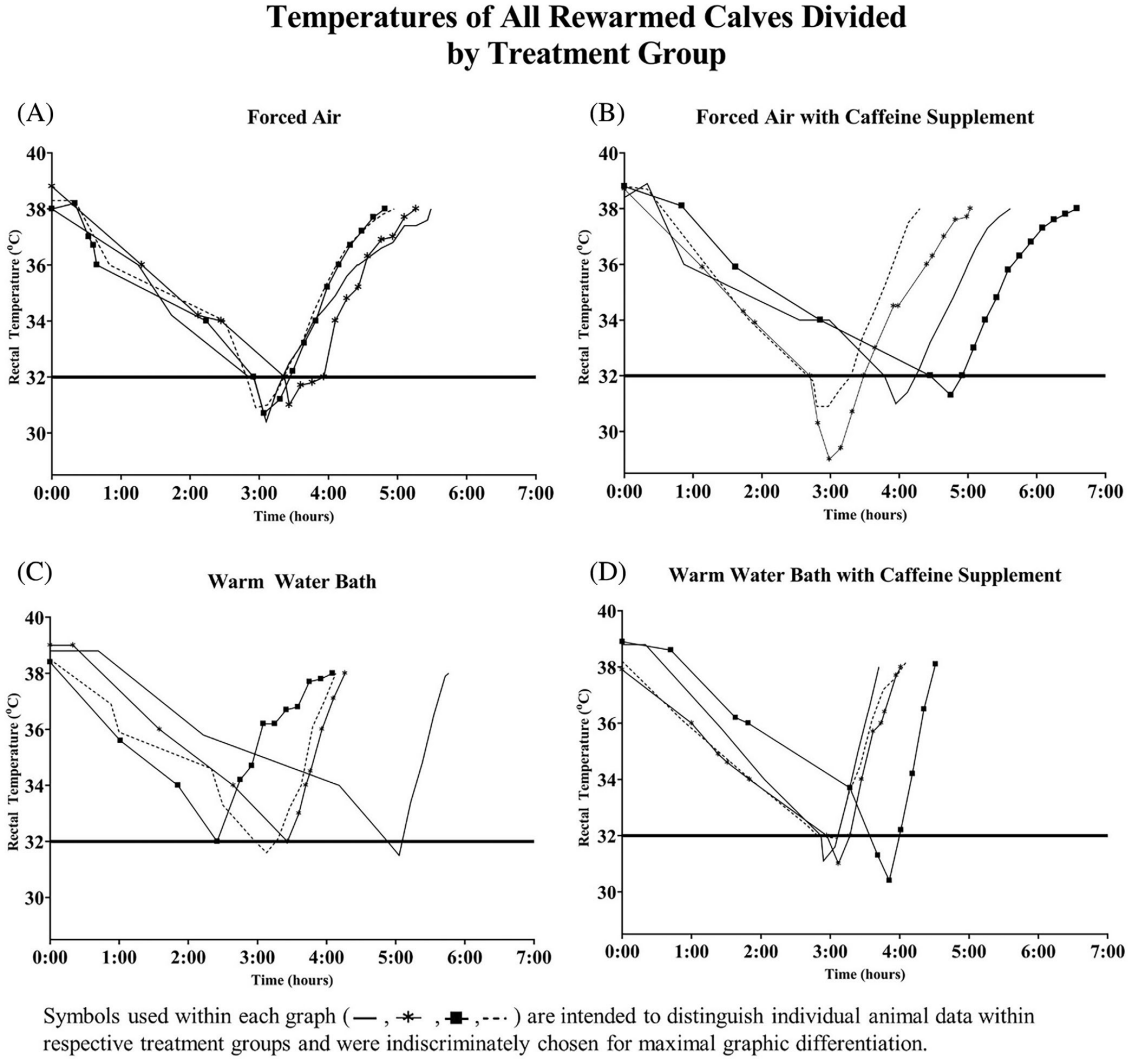
Rectal temperatures graphed over time of all animals divided by treatment groups: Forced air (A), forced air with caffeine supplement (B), warm water bath (C), and warm water bath with caffeine supplement (D).

**FIGURE 2:**
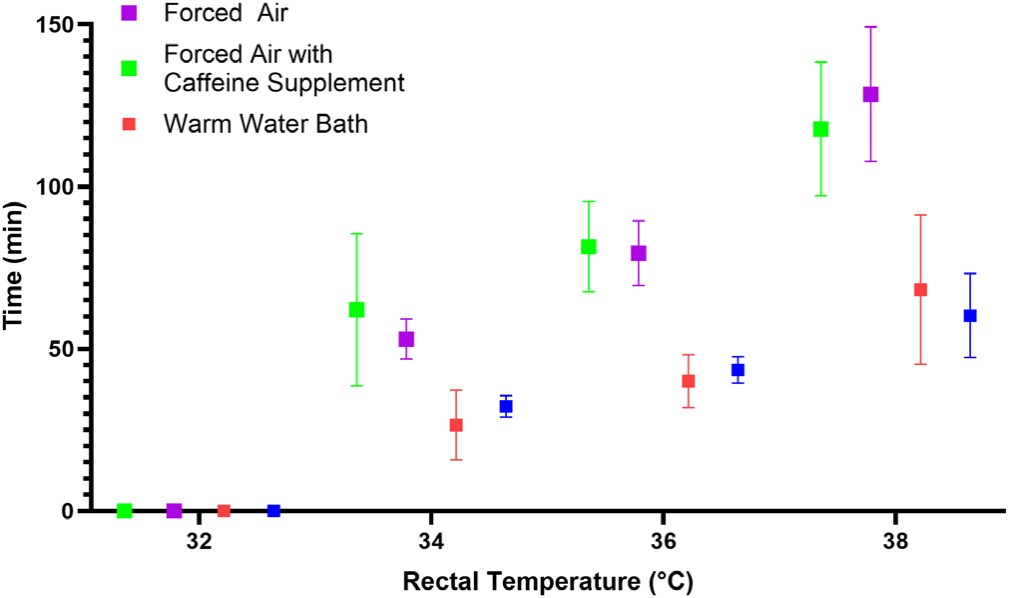
Average time needed to reach benchmark rectal temperatures by treatment during the rewarming period.

Total cooling and rewarming times with respective rates are broken down by cohorts and summarized in Table 1; rectal temperatures over time for all calves by group are presented in Figure 1A–D; average time needed to reach benchmark rectal temperatures by treatment is presented in Figure 2. The rate of rectal temperature decline (0.03°C per minute) and total cooling time (191.0 ± 33.3 minutes) did not significantly differ among treatment groups, regardless of weight. The amount of afterdrop, defined as the body temperature loss after removal from the cool water bath, varied among animals. Collectively, forced air–resuscitated calves experienced greater afterdrop (1.4°C ± 0.7°C) than warm water–treated calves (0.6 ± 0.6°C), with the most severe incidence recorded 3°C below the desired nadir in a forced air–resuscitated calf. The total rewarming time required to return rectal temperatures to 38°C using warm water was significantly faster (0.1°C per minute; 64.3 ± 17.8 total minutes; CI, 0.069‐0.091; *P* < .05) than forced air (0.05°C per minute; 123.1 ± 20.0 total minutes; CI, 0.037‐0.051), regardless of caffeine supplement administration. Adjunct caffeine supplementation did not reduce total rewarming time for both warm water–treated and forced air–resuscitated animals, 8.3 and 10.8 minutes faster, respectively (*P* = .14).

**TABLE 1:** Summary of time spent by each cohort and corresponding temperature change rates broken down by thermal phases.

Group	Total cooling time (min)	Cooling rate (°C/min)	Total rewarming time (min)	Rewarming rate (°C/min)
Forced air only	179.3 ± 15.4	0.3 ± .01	128.5 ± 20.7	0.05 ± .01
Forced air with caffeine supplement	205.3 ± 50.9	0.3 ± .01	117.8 ± 20.6	0.05 ± .01
Water bath only	195.0 ± 44.9	0.3 ± .01	68.3 ± 22.9	0.09 ± .03
Water bath with caffeine supplement	184.8 ± 24.4	0.3 ± .01	60.3 ± 13.0	0.1 ± .02

*Note*: Unless noted, all values are reported as mean ± standard deviation.

The opposite occurred during the warming process, regardless of treatment modality. Only the calves rewarmed in warm water, or warm water with caffeine supplementation, had blood glucose concentrations above baseline at the end of the study. Comparatively, lactate remained increased above baseline across all treatment groups for the study duration once the cooling process was initiated. Peak concentrations of either analyte was not consistently associated with rectal temperature nadirs. For example, only 9 of 16 hypothermic calves had maximum lactate concentrations at 32°C rectal temperature. The remaining peak concentrations were scattered among various temperature points, including the single highest lactate concentration (10.8 mmol/L) identified at 36°C rectal temperature during the cooling period. Peak glucose concentrations for all treated calves occurred between 32°C and 34°C. The single highest recorded glucose concentration (35.6 mmol/L at 32°C) was nearly unchanged from the preceding measurement (35.5 mmol/L at 34°C) in the same calf.

**FIGURE 3:**
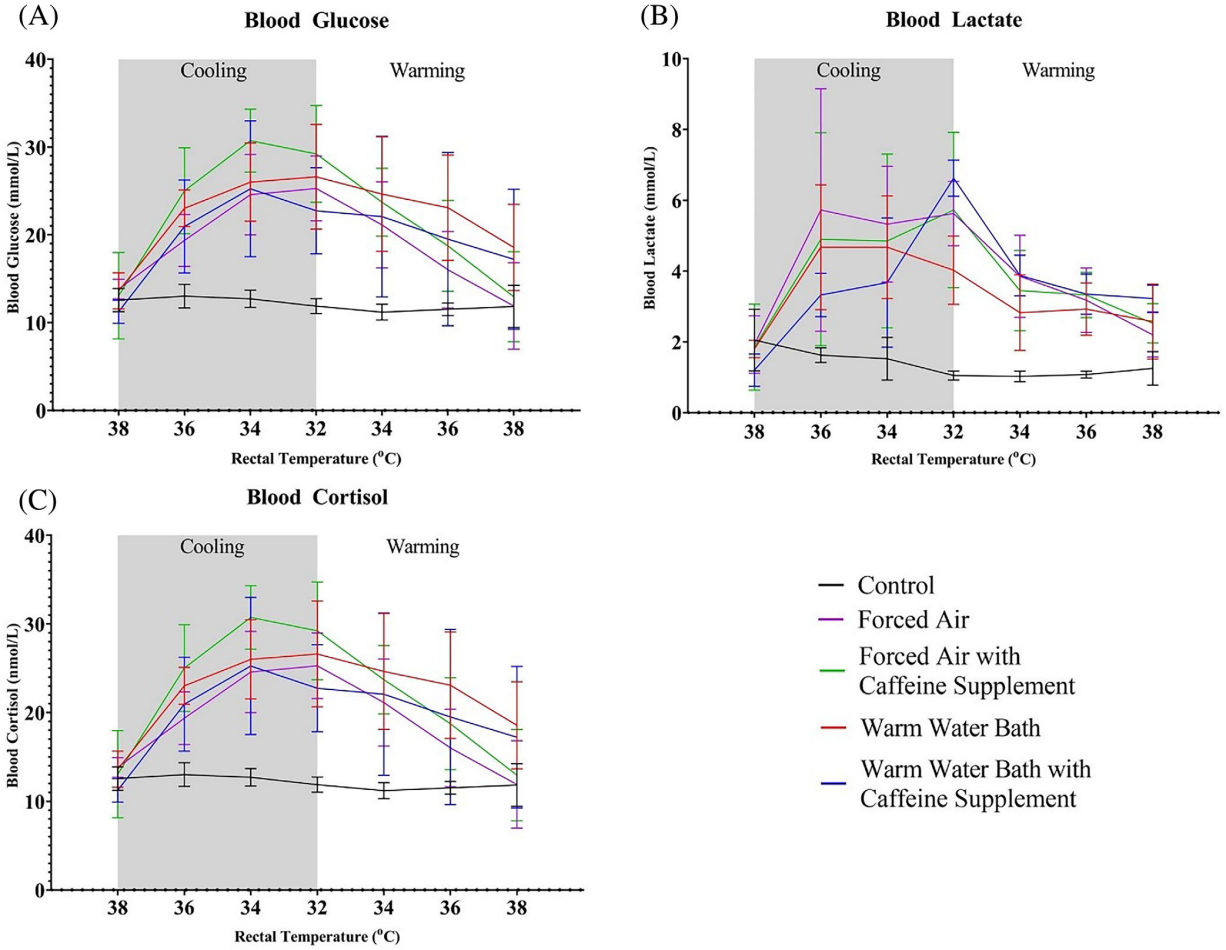
Average blood glucose (A), lactate (B), and cortisol (C), at benchmark rectal temperatures of all calves throughout the study. Gray shaded region of the graph indicates cooling period.

Blood glucose and lactate concentrations are shown in Figure 3AB. Plasma cortisol increased during the cooling process in all calves, peaked at 32°C rectal temperature, and declined during resuscitation in a similar, yet less distinct, inverse relationship with rectal temperature as shown in Figure 3C. Cortisol change rates were not significantly different among treatment groups during either thermal phase. Cortisol concentrations of treated calves were statistically higher than control animals once cooling commenced. Comparison of the single highest cortisol concentration between hypothermic (534.1 nmol/L) and negative control (183.7 nmol/L) calves further illustrates the effect of cooling on cortisol concentrations. Increased calf body mass significantly slowed the change rate of cortisol (*P* = .03) during the rewarming period but affected no other physiologic variable throughout the study.

## 4 DISCUSSION

This study compares total rewarming times of calves resuscitated in warm water baths or forced air delivery systems and reports caffeine use in hypothermic calves. The results confirm that warm water baths increase rectal temperatures faster, and caffeine administration had no detected effects on recovery time. Moreover, inverse relationships between rectal temperature and blood analyte values (glucose, lactate, and cortisol) were observed.

Careful extrication of hypothermic mammals from the inciting environment is essential for preventing further heat loss, cardiac arrythmias,^21^, ^22^ and subsequent deterioration of physiologic biomarker derangements. The most notable consequence of hypothermia, besides death, is the development of irreversible brain damage secondary to hypoxia reported frequently in human literature.^23^ The physiologic similarities between hypothermia and dystocia‐induced hypoxia suggest that hypothermic calves are at a greater risk of neonatal infections and require more intense management because of poor suckle reflex, reduced movement, and decreased colostrum absorption.^24^, ^25^ Successful thermal resuscitation often requires extracorporeal heat supplementation as chilled calves can have a near 50% reduction in metabolic heat production capacity.^8^ Optimal rewarming strategies must reverse the thermal gradient through exploitation of the 4 heat transfer pathways: conduction, convection, radiant exchange, and evaporation. Manipulating just 1 heat transfer method in the natural setting is challenging. Consequently, this study compared the net changes associated with the culmination of heat transference pathways in the respective aqueous and gaseous microenvironments surrounding neonatal calves during a rewarming process.

A circulating cooling bath is an effective method to decrease rectal temperatures in neonatal calves. Time to 32°C rectal temperature (191.0 ± 33.3 minutes) was not significantly different among calves and aligns with values reported in cold‐stressed studies of similar design (172 ± 87 minutes^26^ and 167 ± 7.2 minutes^8^). The current study illustrates that both forced air and warm water baths can successfully rewarm hypothermic neonatal calves. The duration of time needed to return rectal temperatures to 38°C, using the warm water bath, was similar to that reported previously (59 minutes ±6.4) and demonstrates the efficiency and reliability of warm water submersion for resuscitation. These findings are largely attributable to the superior thermal conductivity and specific heat of water over air by a magnitude of 25 and 1000 times greater,^27^ respectively. A shorter time to euthermia could result in a favorable outcome through reduced severity of lesions and fatality rate. However, there is concern in human literature surrounding rapid and uncontrollable peripheral vasodilation. Therefore, recommended thermal resuscitation rates range from 0.25 to 2°C/hour and 2°C/day depending on a patient's comorbidities and whether hypothermia was accidental or medically induced.^28^, ^29^

Certain scenarios might warrant even slower recovery rates as in traumatic brain injury patients because of the neuroprotective nature of controlled hypothermia.^30^ Unpredictable field conditions and scant heat sources unfortunately reduce the ability to monitor and adjust the calf's microenvironment resulting in increased risk of death during recovery. Because all calves in the current study recovered uneventfully and continued to nurse without difficulty, the clinical benefit of acute versus gradual recovery was undetectable. Furthermore, postmortem lesions previously associated with hypothermia, that is, distal limb subcutaneous edema and hemorrhage^26^ were not reported in any calf enrolled in a subsequent, nonrelated terminal clinical trial. The controlled cooling and abrupt initiation of a rewarming process could have prevented such complications, making duration of hypothermic resuscitation equivocal. The paucity of thermal resuscitation guidelines in bovid neonates and the lack of resources in field conditions makes determining a superior recovery method of hypothermic calves challenging.

Aside from the thermodynamic differences between water and air, proposed reasons for the additional time needed to resuscitate calves in the forced air delivery system include the addition of a hair coat–drying phase, unidirectional airflow within the chamber, and afterdrop phenomenon related to excessive calf movement. Because cold water submersion was used in our study to induce hypothermia, all calves resuscitated in the forced air chamber were wet, despite efforts to towel dry each calf. Much of the initial heat imposed by the rewarming system could have been externally consumed through water evaporation within the hair coat, delaying heat transference. The unidirectional heat source within the chamber disproportionately facilitated heat transfer over the skin surface, as evidenced by patchy areas of wet hair upon conclusion of the rewarming period. Although this experimental model could closely reflect the rewarming pattern of cold newborn calves covered in amniotic fluid, it might not represent the rewarming characteristics of dry hypothermic calves resuscitated in a forced air chamber. Nonetheless, these observations, in addition to known variations in tissue rewarming rates,^31^ illuminate the importance of rotating calves within a forced air rewarming unit. The ambient temperature within these units should also be thermostat regulated as maximum temperatures can dangerously exceed 49°C.

Rectal temperatures of calves resuscitated with forced air continued to decline at the onset of rewarming in a similar manner described in human rescue literature as the “afterdrop phenomenon.”^22^, ^23^, ^32^, ^33^ In fact, excess movement after removal expedites heat loss^32^ in humans and might have been apparent in this study. Although the exact mechanism of core temperature loss during this period is controversial,^34^, ^35^, ^36^, ^37^ it is likely because of a combination of thermodynamic processes. With no way to immobilize the calf within the forced air chamber, rectal temperature nadirs were nearly 3°C below the intended end point. A similar degree of afterdrop was not observed in calves rewarmed in water, possibly because of sufficient diffuse heat distribution. The clinical importance of afterdrop during the rewarming process of hypothermic neonatal calves is not known. Nonetheless, extrapolation from human avalanche survival literature suggests providing an immediate heat source as cooling rates can quadruple shortly after rescue.^23^, ^32^, ^34^

Evaluation of circulating cortisol concentration is common practice among food animal stress or pain studies due its prevalent role in the hypothalamic‐pituitary‐adrenal axis.^38^, ^39^ Although basal cortisol concentrations range between 4.4 and 5.0 nmol/L for bull calves,^40^ increases as high as 256.6 nmol/L have been associated with dorsal recumbency at harvest^41^ and 141.46 nmol/L secondary to castration and dehorning.^42^ Initial cortisol concentrations across all animals were increased above the accepted basal concentrations; these results were expected and attributed to transportation, restraint, and catheter placement. Subsequent fluctuations in cortisol observed throughout the cooling and rewarming process were not significant across treatments yet align with previous values in similar neonatal rewarming studies.^11^, ^43^ Peak concentrations observed in some calves (533.9 nmol/L) suggest that hypothermia is physiologically stressful.^44^

Hyperlactatemia has traditionally been associated with increased anaerobic metabolism and often coincides with a poor prognosis for hospitalized humans and veterinary patients of various disease states.^45^, ^46^, ^47^, ^48^ Multiple point of care meters have been developed and provide a relatively quick, inexpensive method for lactate monitoring and are already utilized in treatment decisions of septic and hypovolemic patients. Characterization of lactate trends in this study was intended to provide practitioners with a general understanding of lactate fluctuations in relation to rectal temperature. Because established limits of measurable biomarkers for rewarming rate regulation are lacking, utilizing all available diagnostic modalities, including lactate monitoring, to generate a conclusion is paramount. Shivering, alterations in hemodynamic stability, decreased peripheral tissue perfusion, and epinephrine‐mediated Na‐K‐ATPase pump upregulation likely contributed to the wide range of lactate values seen here (maximum 10.8 mmol/L). Although epinephrine was not measured, supranormal catecholamine blood concentrations have been shown to exist in cold‐stressed neonatal calves.^43^ These results illustrate the importance of serial lactate measurements during treatment as single readings might not correspond to unstable animals.

Hyperglycemia is a common physiologic response to cold stress in animals^49^ and humans^50^ alike, particularly during acute cold exposure; in contrast, prolonged hypothermia typically results in whole body energy depletion and hypoglycemia.^6^, ^51^ Although the change rate of glucose was faster during the rewarming period, there was no difference among groups. Moreover, the mean glucose concentrations at each 2°C interval did not significantly differ. Although postprandial glucose absorption cannot be discounted as a cause of hyperglycemia in the current study, it does not solely explain the temperature‐associated trend seen in treated versus control calves and might not provide enough energy for neonatal thermoregulation.^11^ Similar to the graphic representation of other biomarker trends within the study, plotting glucose against rectal temperature resulted in seemingly more basal euthermic glucose concentrations within forced air rewarmed animals. However, when the differences in time to euthermia among these treatments is taken into consideration, any discernable differences are likely caused by the comparatively longer rewarming period in the forced air cohorts.

The efficacy of various oral adjunct treatments, for example, brandy^8^ and colostrum,^11^ in the management of neonatal calf hypothermia have been evaluated by other studies with underwhelming results. Oral administration of colostrum caused a more profound rectal temperature decline in chilled, newborn Brahman calves compared with IV infusions of glucose,^11^ suggesting that oral energy substrates alone do not improve thermogenesis. Identification of an effective, easily administered thermogenic drug could provide producers with an additional resuscitative measure to reduce hypothermia‐associated mortality. The foundation for oral administration of a caffeine‐containing substance in this study was based on reports suggesting caffeine's ability to upregulate nonshivering thermogenesis in addition to cognitive function and blood pressure modulation.^18^ Caffeine is easily accessible with formulations ranging from tablets to energy drinks.^52^ The effects and adverse events associated with caffeine administration to bovids is underreported, as only 1 study has assessed the pharmacokinetics after IV administration to dairy cattle^53^; no adverse events were reported in that study. The purpose of administration in our study was 2‐fold: evaluate the effect of adjunct caffeine administration on time to euthermia and report any deleterious consequences with oral administration. Although adverse events associated with supplement administration were not recorded, oral administration of any product could prove challenging and dangerous in hypothermic calves because of profound, diffuse muscle rigidity and obtunded mentation. The lethargic mentation of such animals can render them unable to effectively respond to the noxious stimulus of accidental intratracheal esophageal tube placement and result in aspiration pneumonia or drowning. The diffuse muscle rigidity of hypothermic calves, particularly masseter muscles, is uncommonly discussed in the literature. Knowledge of such muscle tone is useful when providing recommendations for on farm hypothermic calf resuscitation.

Limitations of this study include the sample size and the nature of the point of care analyzers used for lactate and glucose observation. Although likely underpowered, similar calf rewarming studies have used cohorts of 4 to 5 ruminant neonates.^8^, ^9^ The glucometer utilized in this study has a demonstrated consistent precision and a consistent bias when compared with a validated for cattle point of care analyzer.^54^

## 5 CONCLUSION

Warm water submersion remains the quickest heat transfer method for restoration of euthermic rectal temperatures in hypothermic neonatal calves. However, forced air delivery systems are a viable alternative when rectal temperatures are at least 30°C.

## CONFLICT OF INTEREST DECLARATION

Authors declare no conflict of interest.

## OFF‐LABEL ANTIMICROBIAL DECLARATION

Authors declare no off‐label use of antimicrobials.

## INSTITUTIONAL ANIMAL CARE AND USE COMMITTEE (IACUC) OR OTHER APPROVAL DECLARATION

Authors declare no IACUC or other approval was needed.

## HUMAN ETHICS APPROVAL DECLARATION

Authors declare human ethics approval was not needed for this study.
